# A cross-sectional study of traditional practices affecting maternal and newborn health in rural Nigeria

**DOI:** 10.11604/pamj.2018.31.64.15880

**Published:** 2018-09-28

**Authors:** Adenike Oluwayemisi Jimoh, Sunday Enema Adaji, Hamdalla Adelaiye, Abiola Aira Olorukooba, Umma Bawa, Habiba Ismail Ibrahim, Comfort Garba, Anita Lukong Mfuh, Suleiman Idris, Sunday Oladapo Shittu

**Affiliations:** 1 Department of Paediatrics, College of Health Science, Bingham University, Jos, Nigeria; 2 Department of Obstetrics and Gynaecology, Ahmadu Bello University, Zaria, Nigeria; 3 Department of Paediatrics, Ahmadu Bello University Zaria, Nigeria; 4 Community Midwife, CBS Research Group, PRHI, Ahmadu Bello University Zaria, Nigeria; 5 Department of Nursing Sciences, Ahmadu Bello University, Zaria, Nigeria; 6 Department of Community Medicine, Ahmadu Bello University Zaria, Nigeria

**Keywords:** Harmful traditional practices, newborn, maternal health, female genital mutilation, early marriage, childbirth, uvulectomy

## Abstract

**Introduction:**

Certain traditional practices which have negative effects on maternal and child health continue to be practiced in sub-Saharan African countries. A survey was carried out in a rural village in Nigeria to understand the scale and range of these practices.

**Methods:**

This was a cross-sectional study in which trained interviewers administered pre-tested questionnaires on child-bearing women using questionnaires embedded on android devices.

**Results:**

The median age of marriage and pregnancy were 15 and 16 years respectively. Home births were high (90.4%) while non-skilled birth attendant was 87.4%. The community had a son preference index ratio of 1:4.1. Up to 81.5% of mothers responded that one form of unhygienic traditional procedure or the other was performed on their children. Time to initiation of breast feeding was in hours in the majority (76.3%) of mothers, with a high rate of use of prelacteal feeds (85.2%). Being an adolescent mother (AOR 0.403, 95%CI 0.203, 0,797) and utilizing a skilled provider at birth (AOR 0.245, 95%CI 0.088, 0.683) were associated with less likelihood of having an unhygienic procedure performed on children.

**Conclusion:**

The findings of our study suggest that traditional practices which could have negative effects on maternal and child health are still ongoing in the study community. Child protection laws and safeguarding principles could help to reduce these practices and would need to be developed and implemented in these settings where these practices are still prevalent.

## Introduction

The prevalence and effects of harmful traditional practices especially in sub-Saharan African countries remains a public health concern. Practices like female genital mutilation, early and forced marriage, early pregnancy and son preference which violate the rights of women and girls continue to generate passion on all sides. The consequences of these practices on maternal and newborn health have been documented in several texts [1, 2]. While efforts intensify in several country settings to eradicate these practices and manage the consequences, some countries continue to struggle with how to develop interventions to eradicate such practices. Multi-ethnic countries like Nigeria face special challenges of multiculturalism and associated practices. Most of these practices have stood the test of time and are often grounded in unfounded beliefs of their benefits. For example, some communities perform female genital mutilation on girls with the belief that it cleanses and makes girls modest. Some cultures encourage early marriage because of beliefs of protection within marriage against sexually transmitted infection and unwanted pregnancies and its consequences [3]. Practices like Uvulectomy have been practiced for centuries for beliefs that they have therapeutic effects [4]. The harmful effects of many of these practices have for long been established. A practice like female genital mutilation has gained worldwide attention as many immigrants from sub-Saharan African countries arrive Europe and North America. Accounts from the literature document disabling and sometimes life-threatening complications associated with FGM [5, 6]. Similar documentation exist of the harmful effects of other unhygienic traditional procedures like uvulectomy and scarification [7]. As for practices like early marriage and pregnancy, preference for home births and male children, there is also a huge amount of literature on the consequences on these practices [8-10]. International organizations like the United Nations have recognized the harmful effects of these practices and emphasized the associated human right violations [11]. The persistence of these practices especially in sub-Saharan countries justifies continued studies to highlight the scale of the problem and keep the conversation going on how best strategies to reduce these practices and mitigate their effects. It can be difficult to measure the prevalence of traditional practices, especially those with harmful effects in settings where such practices may have become normalized and therefore not recognized as harmful. However, an understanding of the range and burden of such practices is an important step towards exploring attitudes towards them and community engagement for change. Data for this study was collected during a survey of maternal and newborn health in rural northern Nigerian community. The study aims to characterize such traditional practices which could have implications on maternal and newborn health. Secondarily we examined individual level and family factors influencing the unhygienic surgical procedures like female genital mutilation performed on newborns as part of tradition. As global efforts gather momentum under the SDGs to improve maternal and child health. It is hoped that the findings would stimulate further interest as well as generate more intense conversations about aspects of culture which could be harmful to maternal and newborn health.

## Methods

### Study design

This was a cross-sectional, total community-based survey in which trained interviewers collected data using hand held devices (mobile phones and tablets).

### Study setting and participants

The study was carried out in Tsibiri village, a rural community in Giwa Local Government area of Kaduna state, north western region of Nigeria. At the time of the study, the community had an estimated population of about 1800 inhabitants dwelling in 248 households. Close to 20% (350) of the population were women in the reproductive age group and girls aged 10-14 years who were married. The community is essentially homogenous with Hausa-Fulani and Islam as the predominant ethnicity and faith respectively. The men were mainly farmers while some of the women were petty traders. The nearest comprehensive emergency obstetric centre (EmOC) was about 10 kilometers away while a regional referral hospital was 20 kilometers away from the community.

### Sample size

The study targeted all women aged 15-49 years and those less than 14 years but who were married. There were a total of three hundred and nineteen (319) women aged 15-49 years and those 10-14 years but who were married.

### Inclusion criteria

All women, aged 15-49 years and those 10-14 years but married, who were members of the community and living in the community at the time of the study and who gave consent or whose husband gave consent were included in the study.

### Ethical consideration

The study was approved by the ethical and scientific committee of the Ahmadu Bello University Teaching Hospital Zaria (the home institution of the research team) and the Institutional Review Board of the Population Council New York (the study funders) (Protocol number 485). Informed consent was also obtained from the community head, each household head and study participants before interviews were conducted.

### Definition of core terms

For the purpose of this study, the following definitions were adopted: Traditional uvulectomy was regarded as a procedure involving the cutting of the uvula and sometimes the near-by structures such as the tonsils, usually by a non-physician or traditional healer. An adolescent was defined as one aged between 10 and 19 years while early marriage referred to marriage or union before the age of 18 years. Prelacteal feeding was defined as the practice of providing a newborn any feed, other than breastmilk before initiation of lactation. Female genital mutilation (FGM): all procedures that involve partial or total removal of the external female genitalia, or other injury to the female genital organs for non-medical reasons. Son preference referred to preference accorded to the boy child over the girl child. The son preference index (SPI) is the measure used to indicate preference for more sons (SPI > 0.5), more daughters (SPI < 0.5) or that ‘sex does not matter’ (SPI = 0.5). A skilled birth attendant (SBA) was defined as “an accredited health professional such as a midwife, doctor or nurse who has been educated and trained to proficiency in the skills needed to manage normal (uncomplicated) pregnancies, childbirth and the immediate postnatal period, and in the identification, management and referral of complications in women and newborns”

### Data collection and devices

Hand held devices running on Android Operating System pre-installed with Open Data Kit (ODK) Data Collect application were used for data collection. The survey questionnaire was adapted from the Nigerian 2008 Demographic and Health Survey questionnaire and formatted into ODK compatible software and uploaded into the devices. Each household was allotted a number for identification. A manual map of the community with all households was sketched and a GPRS co-ordinate of each household was also obtained. Using the hand held devices, 6 female interviewers who had been trained in the use of the devices administered the questionnaire over a period of 5 days in 2011 with each interview lasting 15 – 20 minutes. Pre-testing was carried out prior to eventual field work. After each interview, the data collected was sent wirelessly to a server on which the data was aggregated and subsequently downloaded.

### Data analysis

The data were cleaned, coded and checked for missing values and outliers. Data were analyzed using statistical software SPSS version 20.0.0. Simple percentages, descriptive measures were used to represent results obtained. Binary logistic regression was applied to understand the strength of the relationship between selected sociodemographic variables and unhygienic traditional procedures carried out on children. Son preference index (SPI) was calculated by the formula: Desired number of sons/desired number of children.SPI greater than 0.5 = preference for sons. SPI equal to 0.5 = “sex doesn’t matter” while SPI measure less than 0.5 signifies girl preference.

## Results

### Socio-demographic characteristics

There were 319 women of reproductive age group including 10-14 year old girls who were married in the community. Of these 309 (96.9%) were available and were interviewed. The socio-demographic characteristic of the study population is as shown in Table 1.

**Table 1:** Sociodemographic characteristics of the study population

Characteristics	Frequency (N=309)	Percentage (%)
**Age group(years)**		
10-19	77	24.9
20-29	135	43.7
30-39	81	26.2
40+	16	5.2
**Marital status**		
Single	10	3.2
Married	299	96.8
**Educational level of women**		
No formal education	178	57.6
Primary	82	26.5
Secondary	22	7.1
Tertiary	27	8.7
**Educational level of husbands(n=299)^§^**		
No formal education	144	48.1
Primary	55	18.4
Secondary	72	24.1
Tertiary	28	9.4

§ some women were in polygamous setting, hence number of husbands less than number of women

### Young age of marriage and pregnancy

The median age of first marriage was 15 years while the median age of first pregnancy was 16 years. Details are as shown in Table 2.

**Table 2:** Young age of marriage and pregnancy

Age (years)	Frequency (n)	Percentage (%)
**First marriage (n=299)**		
10-14	134	44.8
15-19	159	53.2
20-24	6	2.0
**First pregnancy(n=276)**		
10-14	41	14.9
15-19	213	77.2
20 and above	22	7.9

### Home births and delivery by unskilled birth attendants

As shown in Table 3, the majority of women had home births and utilized unskilled providers during their recent childbirth.

**Table 3:** Preference for home births and unskilled birth attendants

Place of delivery (N=270)^†^	Frequency	Percentage
Home	244	90.4
Health facility	26	9.6
**Birth Attendant at Delivery (n=270)^†^**		
No birth attendant	182	67.4
Non-skilled birth attendant	54	20.0
Skilled birth attendant	34	12.6

### Preference for the male child

As shown in Table 4, sons were preferred to daughters with a son to daughter preference ratio of 1.4:1.

**Table 4:** Preference for male children

Son preference (n=267)	Frequency (n)	Percentage (%)
Son preference index > 0.5	70	26.2
Son preference index < 0.5	50	18.7
Son preference index = 0.5	147	55.1
Total	**267**	**100**

† Included only those who had their last child birth within 24 months prior to the survey

### Delayed initiation of breastfeeding and use of prelacteal feeds

The majority of the respondents (84.8%) breastfed the newborns after their most recent childbirth. However as shown in Table 5, initiation of breastfeeding was delayed in the majority of cases with the use of prelacteal feeds by most mothers to feed their newborns.

**Table 5:** Time to initiate breast feeding and use of prelacteal feeds

Time to initiation of breastfeeding (n = 262)	Frequency	Percent
Within a day	56	21.3
Within hours	144	55
Immediately	62	23.7
Total	262	100
**Use of prelacteal feed (n = 270)**		
Yes	230	85.2
No	40	14.8
**Total**	**270**	**100**

### Unhygienic surgical procedures

The majority of the mothers (81.5%) responded that one form of unhygienic surgical procedure or the other was performed on their newborns. The different procedures and their frequencies are as in Table 6.

**Table 6:** Practice of unhygienic traditional surgical procedures (n = 220)

Procedure	Frequency	Percentage
Uvulectomy only	94	42.7
Scarification marks only	9	4.1
Female genital mutilation (FGM) only	1	0.4
Uvulectomy and scarification	27	12.3
Uvulectomy and FGM	60	27.3
All types	29	13.2
**Total**	**220**	**100**

### Individual and family factors influencing unhygienic surgical procedure performed on children

In a binary regression model, we assessed selected individual level and family factors influencing the decision to perform unhygienic procedures (dependent variables) on babies. The variables selected were age at last child birth < 20years, birth attendant during delivery, place of delivery, woman’s schooling and husband’s schooling (independent variables). As shown in Table 7, young age at childbirth as well as being assisted by a skilled provider at birth were protective factors.

**Table 7:** Binary regression analysis of association between selected variables and practice of unhygienic traditional surgical procedures

Selected variable	p-value	Adj OR	95% CI
Age at last childbirth < 20 years	0.009	0.403	0.203, 0.797
Skilled provider at birth	0.007	0.245	0.088, 0.683
Place of delivery - home	0.739	1.219	0.381, 3.902
Woman’s formal schooling	0.842	1.079	0.510, 2.284
Husband’ schooling	0.680	0.862	0.426, 1.745

## Discussion

Our findings in this village setting suggest the occurrence of different forms of traditional practices which could have harmful maternal and newborn consequences. Early age at marriage and child bearing, preference for home delivery and male children, delayed onset of breast feeding and prelacteal feeding practice as well as unhygienic traditionally performed surgeries were among traditional practices found in this community. The practice of early age of marriage and child bearing found in our study in not new; similar findings and the health consequences have previously been documented in low and medium income country settings [12, 13]. The factors driving this practice have been the topic of many studies. Such factors include belief that early marriage would preserve the virtue of girls and family honor [14], fulfillment of needs, obedience to parents, low decision making power of girls and bowel to gender expectations [15]. This practice however carries the risk of poor educational development of the girl-child [16], early childhood development and stunting [17], preterm delivery and delivery of low birth weight babies [10]. Studies have also linked early marriage to domestic violence [9] and increased risk of HIV transmission [18]. The health benefit of delaying the age of marriage and child bearing has also been a topical issue. In a large study by Delprato and Akyeampong, delaying the age of marriage is beneficial in terms of improving health seeking behaviour for utilization of maternal and newborn health care services [19]. Under the SDGs it would be reasonable to step up efforts to bring this practice to an end as part of the strategies to improve maternal and newborn health.

Home delivery and births by unskilled providers were also identified as a prevalent traditional practice in the study community. Some studies have described these preferences as being rooted in culture and tradition [20, 21]. Certain traditions that require husband’s permission for skilled birth attendant utilization tend to reinforce the preference for home births [22]. Home births and its consequences continue to attract controversies even in high income countries where the movement for planned home births keeps evolving. While some studies conclude that home births are safe [23], others worry about newborn risks [24]. In the context of rural communities with no frameworks and pathways for planned home births, unsupervised home births could have calamitous outcomes. It is therefore important to continue to engage with such rural communities about how to amend these risky traditions meaningfully towards making pregnancy and childbirth safer. The tradition of son preference and its implications has been described in many cultures and countries [25, 26]. The findings of our study corroborate the narrative that son preference continues to be an issue in many country settings [27, 28]. Son preference could be a deciding factor in women’s health as they have been shown to influence fertility and reproductive behavior [29, 30]. In our study context where fertility remains high and contraceptive utilization very low, this issue is particularly relevant as the risks to mothers and newborns could be heightened in the endeavor to fulfill tradition.

Pertaining to unhygienic surgical procedures like female genital mutilation, the findings of the study demonstrates the pervasiveness of these customs in low and middle income, as well as high income countries where it is largely driven by migration [31, 32]. Uvulectomy has also been described in several studies [33]. These procedures could be complicated by bleeding and infection during childhood and in the case of female genital mutilation could lead to sexual and reproductive challenges including sexual difficulties and childbirth complications [34, 35]. Our finding that women aged less than 20 years (i.e. adolescents) compared to older women were less likely to have unhygienic procedure performed on their babies is difficult to explain. It might be that being young mothers, they may be less willing to expose their newborn to such procedure. It might also be that these young women were the ones that utilized skilled birth attendants for during childbirth. A protective effect of skilled birth utilization against traditionally performed unhygienic procedure was demonstrated in our study. This could suggest that women who were delivered by a skilled provider probably benefitted from messages about healthy childcare practices and the risks of such procedures. One Nigerian study showed that utilizing a hospital for health care had a protective effect against female genital mutilation [36]. Although this association was not statistically significant in our study, mothers who had hospital births were likely to allow unhygienic procedures to be performed on their children.

With regards to feeding practices, the majority of respondents delayed initiation of breast feeding and used prelacteal feeds. Delayed breast feeding and use of prelacteal feeds are well known infant feeding issues in some communities in Asia and Africa with culture being cited in some instances as the motivation for this practice [37, 38]. The use of prelacteal feeding can delay onset of breastfeeding [39, 40] or cause childhood morbidity on its own [37]. The negative consequences of delayed initiation of breastfeeding are well known with studies showing an association between delayed breastfeeding and infant morbidity [41]. In the Neovita study early initiation of breastfeeding was found to reduce neonatal and early infant mortality [42]. In the rural setting of our study, the implications of delayed breast feeding and prelacteal feeding might even be more severe due to poor socioeconomic circumstances of mothers. Any strategy to improve newborn health would need to address this practice. Being a cross-sectional study which relied on mothers’ recollection was a limitation of the study. However, our findings were consistent with previous findings from that region of Nigeria. Moreover some of the practices may not be attributed to culture and tradition alone but may be linked to other social and economic factors. For example preference for home births may be linked to poverty, lack of transportation and attitude of health workers as noted by Ashimi *et al* [20]. The desire to have children early and avoid infertility and its associated stigma has been linked to early marriage and childbirth practices in one Pakistani study [43]. Despite these limitations, it is our view that our findings could validly contribute to knowledge on where these practices still occur and the need to address them as part of strategies to improve maternal and child health.

## Conclusion

Our findings show the range and prevalence of different traditional practices which could be harmful to mothers and their children. These findings may not be new. However, they suggest the persistence and pervasiveness of these harmful practices which continue to act as an impediment to optimal maternal and child health in setting where they are practiced. Cultural practices, because of their deep roots in beliefs and perceptions may be difficult to tackle. Child protection laws would need to be enacted and safeguarding frameworks developed and institutionalized to reduce these traditional practices which are inimical to the health of mothers and their newborns.

### What is known about this topic

- Traditional practices abound across sub-Saharan Africa and they have stood the test of time;
- The negative consequences of the harmful traditional practices have been identified and documented;
- Harmful traditional practices act as impediments to optimal maternal and child health where they are practiced.

### What this study adds

- The study emphasizes the need to measure the scale of existence as this is the only way to know how well it is being tackled or not;
- The study brought out the range and burden of harmful traditional practices in a rural northern Nigerian community;
- The findings showed the persistence and pervasiveness of these practices and a need to enact maternal and child protection laws in order to reduce the burden.

## Competing interests

The authors declare no competing interests.
